# cAMP Signaling Regulates Synchronised Growth of Symbiotic *Epichloë* Fungi with the Host Grass *Lolium perenne*

**DOI:** 10.3389/fpls.2016.01546

**Published:** 2016-10-27

**Authors:** Christine R. Voisey, Michael T. Christensen, Linda J. Johnson, Natasha T. Forester, Milan Gagic, Gregory T. Bryan, Wayne R. Simpson, Damien J. Fleetwood, Stuart D. Card, John P. Koolaard, Paul H. Maclean, Richard D. Johnson

**Affiliations:** ^1^Forage Science, AgResearch Ltd., Grasslands Research CentrePalmerston North, New Zealand; ^2^Formally of Forage Improvement, AgResearch Ltd., Grasslands Research CentrePalmerston North, New Zealand; ^3^Biotelliga Ltd., Institute for Innovation in BiotechnologyAuckland, New Zealand; ^4^Bioinformatics and Statistics Team, AgResearch Ltd., Grasslands Research CentrePalmerston North, New Zealand; ^5^Bioinformatics and Statistics Team, AgResearch Ltd., Lincoln Research CentreChristchurch, New Zealand

**Keywords:** *Epichloë festucae*, *Lolium perenne*, symbiosis, cAMP signaling, reactive oxygen species, hyphal branching

## Abstract

The seed-transmitted fungal symbiont, *Epichloë festucae*, colonizes grasses by infecting host tissues as they form on the shoot apical meristem (SAM) of the seedling. How this fungus accommodates the complexities of plant development to successfully colonize the leaves and inflorescences is unclear. Since adenosine 3′, 5′-cyclic monophosphate (cAMP)-dependent signaling is often essential for host colonization by fungal pathogens, we disrupted the cAMP cascade by insertional mutagenesis of the *E. festucae* adenylate cyclase gene (*acyA*). Consistent with deletions of this gene in other fungi, *acyA* mutants had a slow radial growth rate in culture, and hyphae were convoluted and hyper-branched suggesting that fungal apical dominance had been disrupted. Nitro blue tetrazolium (NBT) staining of hyphae showed that cAMP disruption mutants were impaired in their ability to synthesize superoxide, indicating that cAMP signaling regulates accumulation of reactive oxygen species (ROS). Despite significant defects in hyphal growth and ROS production, *E. festucae* Δ*acyA* mutants were infectious and capable of forming symbiotic associations with grasses. Plants infected with *E. festucae* Δ*acyA* were marginally less robust than the wild-type (WT), however hyphae were hyper-branched, and leaf tissues heavily colonized, indicating that the tight regulation of hyphal growth normally observed in maturing leaves requires functional cAMP signaling.

## Introduction

Temperate grasses such as *Lolium perenne* (Poaceae, subfamily Pooideae) often host mutualistic endophytic fungi in the genus *Epichloë* of the family Clavicipitaceae (Christensen and Voisey, 2009; Card et al., 2014; Leuchmann et al., 2014; Simpson et al., 2014). This genus became prominent when these endophytes were proven to be agents of chronic circulatory and neurological disorders in livestock feeding on infected forage (Bacon et al., 1977; Ball and Prestidge, 1993; Leuchmann et al., 2014). Several classes of endophyte metabolites with mammalian and insect toxicity, as well as invertebrate deterrent effects, have been characterized (Schardl and Phillips, 1997; Schardl et al., 2007, 2013). *Epichloë* infection can also elevate the tolerance of grasses to certain abiotic stresses (Arachevaleta et al., 1989; Malinowski and Belesky, 2000; Vázquez-de-Aldana et al., 2013). Given these attributes, these fungi are often prevalent in native grass habitats, but are also essential for persistence of forage in managed pastoral farming systems where insect pressure is high, as in New Zealand, Australia, and the Americas (Johnson et al., 2013a).

*Epichloë* hyphae within host tissues are confined to the intercellular spaces and do not invade cells (Christensen et al., 2008). They are notable for their complex biotrophic lifecycle which is synchronized with growth and development of the host from seedling to mature plant. Colonization of the plant by the endophyte proceeds through discrete modes of hyphal growth that alternate between apical extension and branch formation of hyphae in the shoot apical meristem (SAM), intercalary hyphal growth along the length of the filament in expanding host structures, and a phase in mature plant tissues where the fungus stops growing but remains metabolically active (Christensen et al., 2008; Christensen and Voisey, 2009; Voisey, 2010; Eaton et al., 2011). Each phase of vegetative hyphal development is seemingly initiated in response to changes in host development. For example, the transition between plant cell division and extension in developing leaves correlates with repression of hyphal lateral branch formation and initiation of intercalary hyphal extension; and maturation (cessation of growth) of host leaves correlates with a transition from hyphal intercalary growth to little or no extension or tip (polar) growth. The developmental switch from polar to intercalary hyphal growth is a critical stage in host colonization, and is achieved through initiation of the full cell cycle within intercalary hyphal compartments, including mitosis, the laying down of new septa, and cell expansion, a mechanism of growth rarely observed in vegetative filamentous fungi (Christensen et al., 2008; Christensen and Voisey, 2009; Voisey, 2010). Growth of *Epichloë* hyphae in plants is therefore restricted to developing plant tissues, particularly those arising from the SAM, axillary meristems (from which new tillers form) and floral meristems; and plant structures undergoing cell expansion such as developing leaves and floral spikes. How *Epichloë* colonization processes are synchronized with host development is likely mediated through hyphal sensing of changes in host development that induce corresponding changes in fungal development. *E. festucae* genes in the stress-activated mitogen-activated protein kinase (*sakA*) (Eaton et al., 2010), pH-sensing (*pacC*) (Lukito et al., 2015) and striatin-interacting phosphatase and kinase complex (*mobC*) (Green et al., 2016) are required for regulation of hyphal growth in *L. perenne*, and their deletion induces aberrant hyphal distribution in plants and alters host growth and development. Production of reactive oxygen species (ROS) by *E. festucae* in culture and *in planta* is also vital for establishment of normal symbiotic associations between these organisms. Superoxide ions regulate many processes in fungal morphogenesis and growth, and deletion of *E. festucae* genes encoding proteins of the NADPH oxidase complex responsible for superoxide synthesis, including *noxA, noxR, racA*, and *bemA* also disrupts the phenotype of both the endophyte and the host during symbiosis (Takemoto et al., 2006, 2011; Tanaka et al., 2006, 2008).

The ubiquitous signaling molecule, adenosine 3′5′- cyclic AMP (cAMP) is an integral component of signaling in most organisms. Accumulation of cAMP in the cytoplasm is modulated through the activities of adenylate cyclase (AC) and phosphodiesterase enzymes that synthesize or degrade cAMP respectively. The main target of cAMP in fungi is cAMP-dependent protein kinase (PKA) which mediates many of the physiological effects (D'Souza and Heitman, 2001) by phosphorylating target proteins such as protein kinases, ion channels, and transcription factors. Recently however, other unidentified target(s) of cAMP have been detected, such as those shown to be involved in the regulation of the *Fusarium fujikuroi* secondary metabolite, fusarubin (Studt et al., 2013). To date fungi have been shown to possess a single adenylate cyclase gene that encodes a large membrane-bound enzyme that is stimulated by a variety of environmental signals, and acts down-stream of heterotrimeric G proteins (Ivey and Hoffman, 2005; Kamerewerd et al., 2008) and CO_2_ or ${\text{HCO}}_{3}^{-}$ (Bahn and Mühlschlegel, 2006; Mogensen et al., 2006). This versatility contrasts with mammalian cells that possess several adenylate cyclase enzymes, both cytosolic and plasma-membrane localized, each responding to specific stimuli (McDonough and Rodriguez, 2012). The implication of this is that the single fungal AC enzyme has many interaction partners and is highly interconnected with other pathways to mediate its effects.

Although highly conserved across the fungal kingdom, the components of the cAMP-PKA signaling pathway regulate functionally diverse processes including hyphal growth, secondary metabolite biosynthesis (García-Martínez et al., 2012), conidiation (Mukherjee et al., 2007), reaction to oxidative stress (Choi and Xu, 2010; Deveau et al., 2010), and virulence (Kohut et al., 2010; García-Martínez et al., 2012; McDonough and Rodriguez, 2012). Disruption of AC can have opposing effects in different fungi, even in closely related species (McDonough and Rodriguez, 2012). For example, in *F. fujikuroi*, deletion of the adenylate cyclase gene increases colony sensitivity to oxidative stress (García-Martínez et al., 2012) while in *F. proliferatum* and *F. verticillioides* the mutants are less sensitive than wild-type (Choi and Xu, 2010; Kohut et al., 2010). Similarly, disruption in cAMP signaling has no impact on virulence of *F. fujikuroi* on tomato (García-Martínez et al., 2012), while in *F. proliferatum* virulence on tomato and maize is reduced (Kohut et al., 2010). Generally however, AC is indispensable for virulence in many pathogenic fungi, or nearly so (Klimpel et al., 2002), including the pathogens of insects (Liu et al., 2012), fungi (Mukherjee et al., 2007), humans (Bahn and Sundstrom, 2001; Brakhage and Liebmann, 2005) and plants (Kulkarni and Dean, 2004; Martínez-Espinoza et al., 2004; Yamauchi et al., 2004; Mukherjee et al., 2007; Choi and Xu, 2010; Kohut et al., 2010; Bormann et al., 2014). Reduction in virulence in AC pathway deletion mutants is largely due to the pleiotropic effects of cAMP on the development and functionality of specialized infection structures (Adachi and Hamer, 1998; Yamauchi et al., 2004) or alterations in secondary metabolite biosynthesis (Brakhage and Liebmann, 2005; Sugui et al., 2007; Gallagher et al., 2012) and other virulence factors (Alspaugh et al., 2002).

The cAMP pathway is also important in fungal growth and development. Deletion of the adenylate cyclase gene reduces radial growth and produces a more compact colony in *F. verticilloides, F. proliferatum* and *F. fujikuroi* (Choi and Xu, 2010; Kohut et al., 2010; García-Martínez et al., 2012). Cyclic-AMP also regulates the transition from yeast to filamentous forms in *Candida albicans* (Xu et al., 2008), *Ustilago maydis* (Martínez-Espinoza et al., 2004), and *Paracoccidioides brasiliensis* (Chen et al., 2007) and can either increase (Kohut et al., 2010) or decrease (Choi and Xu, 2010) production of conidia, and delay conidial germination (Kohut et al., 2010).

Despite extensive investigation of the multiple processes influenced by cAMP signaling in pathogens, the role of this pathway in regulating colonization and symbiosis by mutualistic fungi is currently unknown. Here we describe the role of cAMP signaling in the establishment and maintenance of a symbiotic partnership between *E. festucae* and the temperate grass species *Lolium perenne* (perennial ryegrass). We report on disruption of the adenylate cyclase gene (herein designated *acyA*) and consequent defects in hyphal growth and morphology in colonies growing in axenic culture, and the requirement for functional cAMP signaling in *E. festucae* for accumulation of ROS. We also show that *E. festucae* cAMP signaling, unlike most pathogens, appears to modulate growth of the fungus in plants, limiting over-colonization and enabling the symbionts to grow synchronously with plants during development.

## Materials and methods

### Fungal strains

The adenylate cyclase gene (*acyA*) was originally cloned and sequenced from *E. festucae* var. *lolii*, previously *Neotyphodium lolii* (Leuchmann et al., 2014), strain Lp19, isolated from *L. perenne*. Gene disruption experiments were performed on the closely related strain *E. festucae* Fl1, isolated from *Festuca trachyphylla* (Hack.) Krajina. The strains used in this study are presented in Table 1.

**Table 1:** ***E. festucae* strains used in this study**.

**Strain**	**Genotype**	**References**
*E. festucae var. lolii* Lp19	wild type	Christensen et al., 1993
*E. festucae* Fl1	wild type	Leuchtmann, 1994; Moon et al., 1999
*E. festucae* Fl1 EGFP	*pTef::EGFP::GA; hph*	Christensen et al., 2008
*E. festuae* Fl1 *acyA19*	Ectopic insertion of acyA	This study
*E. festuae* Fl1 *acyA49*	Ectopic insertion of acyA	This study
*E. festuae* Fl1 Δ*acyA*34	Δ*acy::hph*	This study
*E. festuae* Fl1 Δ*acyA*42	Δ*acy::hph*	This study
*E. festuae* Fl1 Δ*acyA*47	Δ*acy::hph*	This study
*E. festuae* Fl1 Δ*acyA*34/*acyA*	Δ*acy::hph; acyA, gen*	This study
*E. festuae* Fl1 Δ*acyA*42/*acyA*	Δ*acy::hph; acyA, gen*	This study
*E. festuae* Fl1 Δ*acyA*42/EGFP	Δ*acy; hph; pTef::EGFP::GA, gen*	This study

### Fungal and plant growing conditions

Fungi were cultured on potato dextrose agar (PDA, Difco, Le Pont, De Claix, France) at 22°C in an 8 h light, 16 h dark cycle. *L. perenne* cv. Nui or Samson plants infected with *E. festucae* Fl1 wild-type or mutant strains were maintained in glasshouse conditions under ambient light and temperature.

### Identification and sequencing of *Epichloë acyA* genes

We previously cloned and sequenced a 300 bp *acyA* PCR product from *E. festucae* var. *lolii* strain Lp19 (Johnson et al., 2007). The DNA fragment was used as a probe to screen an Lp19 genomic DNA lambda library (Fleetwood et al., 2007) to recover a larger fragment of the gene for functional analysis. A single lambda clone (designated 6163) with homology to the adenylate cyclase gene fragment was recovered (Genbank accession KR815911). Sequencing of the 6047 bp insert indicated the presence of 5819 bp of the adenylate cyclase open reading frame (ORF) plus 228 bp of the *acyA* 3' region. The first 1419 bp of the open reading frame was missing. This sequence was used to design the gene disruption vector. Later, *de novo* sequencing of the *E. festucae* Fl1 genome by Schardl et al. (2013) enabled a complete genomic *acyA* sequence (gene model Fl1M3.048730, http://www.endophyte.uky.edu/) to be recovered. BLASTn was used to recover the *acyA* gene from other haploid *Epichloë* species (Table 2).

**Table 2:** **Copy number of *acyA* in haploid and allopolyploid *Epichloë* species**.

**Species**	**Parental species**	**Strain**	**No. of genomes**	***acyA* copy number**	**Genome References**
*E. festucae*	n/a	E2368	1	1	Schardl et al., 2013
*E. festucae*	n/a	Fl1	1	1	Schardl et al., 2013
*E. brachyelytri*	n/a	E4804	1	1	Schardl et al., 2013
*E. glyceriae*	n/a	E277	1	1	Schardl et al., 2013
*E. amarillans*	n/a	ATCC 200744	1	1	Schardl et al., 2013
*E. typhina*	n/a	ATCC 200736	1	1	Schardl et al., 2013
*E. typhina*	n/a	E5819	1	1	Schardl et al., 2013
*Epichloë* sp.	*E. baconii/amarillans* x *E. bromicola*	AR3046^*^	2	2	unpublished
*E. uncinata*	*E. typhina* x *E. bromicola*	AR1006^*^	2	2	unpublished

* allopolyploid strains, n/a is not applicable.

The *acyA* genes from allopolyploids AR3046 (*E. baconii/amarillans* × *E. bromicola*) and AR1006 (*E. typhina* × *E. bromicola*) were recovered by mapping their genome read pairs (unpublished) to 7 kb genomic scaffolds (containing the *acyA* gene) of extant strains of their parental species using the “aln” algorithm BWA version 0.7.9a-r786. For AR3046, 43,768,368 78 bp read pairs with an insert size of ~290 bp were individually mapped to *E. baconii* strain ATCC 200745 and *E. bromicola* strain AL0434 (Schardl et al., 2014). For AR1006, 21,293,733 100 bp read pairs with an insert size of ~160 bp were mapped to *E. typhina* strain ATCC 200736 and *E. bromicola* strain AL0434. The mapped reads were extracted using SAMtools (Li et al., 2009) version 0.1.19-44428cd and imported into Geneious V8.1 (Biomatters, http://www.geneious.com) (Kearse et al., 2012) and consensus sequences for each mapping file were generated based on a threshold of 95% identity.

### Vector construction

The *acyA* disruption vector (pCRVacyhph) was constructed using the Multisite Gateway system (ThermoFisher Scientific, Walden, MA, USA) following the manufacturer's instructions. The entry vector pDONR221/hygromycin, containing the *hph* cassette from pAN7-1 (Punt et al., 1987), was constructed as previously described (Fleetwood et al., 2007). Two further Multisite Gateway entry vectors, pDONRP2R-P3/AC3′ and pDONRP4-P1R/AC5′ containing a 5′ (3002 bp) and 3′ (3064 bp) region respectively, flanking the integration site in *acyA*, were created. PCR products were amplified from *E. festucae* var. *lolii* Lp19 genomic DNA using primer pairs AC5′-attB4 and AC5′-attB1 (for the 5′ flank) and AC3′-attB2 and AC3′-attB3 (for the 3′ flank) using Platinum Pfx DNA polymerase (ThermoFisher Scientific) according to the manufacturer's instructions. Primer sequences are listed in Supplementary Table 1. The PCR products were purified using the QIAquick PCR Purification Kit (Qiagen, Hilden, Germany), and the products quantified by fluorometric quantitation using the Qubit system (ThermoFisher Scientific). The PCR fragments were then recombined into Gateway donor vectors pDONRP4-P1R and pDONRP2R-P3 using Gateway BP Clonase II. The resulting vectors, pDONRP4-P1R/AC5′and pDONRP2R-P3/AC3,′ along with pDONR221/hygromycin, were then recombined into the destination vector pDESTR4-R3 using Gateway LR Clonase II Plus, to create pCRVacyhph.

### Disruption of *E. festucae acyA*

Gene disruption experiments were originally designed to disrupt *acyA* in strain *E. festucae* var. *lolii* Lp19, however as this strain proved to be relatively intractable to homologous recombination the closely-related strain *E. festucae* Fl1 was used instead. Homologous recombinants were obtained by PEG-mediated transformation of protoplasts. Protoplasts were prepared using the method of Young et al. (1998), except that 10 mg/ml of Glucanex (InterSpex Products, San Mateo, CA, USA) was used to digest cell walls for 3 h at 30°C with shaking (100 rpm). *E. festucae* Fl1 was transformed using 5 μg of each plasmid by the method of Vollmer and Yanofsky (1986) with modifications (Itoh et al., 1994). Protoplasts were co-transformed with plasmid pCRVacyhph (see above) or pTEFEGFP (EGFP fused to the *tef2* promoter from *Aureobasidium pullulans*) (Vanden Wymelenberg et al., 1997) plus either pPN1688 (Young et al., 2005) or pII99 (Namiki et al., 2001) for resistance against hygromycin B or geneticin (both Gibco, ThermoFisher Scientific) respectively. Transgenic colonies were selected on PDA containing 150 μg/mL or 200 μg/mL of hygromycin B or geneticin respectively, and regenerating colonies were purified to homogeneity by sub-culturing hyphal tips onto fresh selective media three times. A number of colonies were analyzed by Southern-blot hybridization to confirm that each contained a single integration of the hygromycin resistance cassette at the desired locus. Genomic DNA was extracted from putative recombinant strains using the method of Byrd et al. (1990) and 2 μg of DNA was digested to completion with *Hin*dIII. The DNA was subjected to standard agarose gel (0.8% w/v) electrophoresis, transferred to nylon (Hybond N^+^, GE Healthcare, Buckinghamshire, UK) and the membrane hybridized independently against two dideoxygenin-labeled probes following the manufacturer's instructions (Roche, Basel, Switzerland). Probe 1 (482 bp) was amplified by PCR using the TripleMaster polymerase system (Eppendorf, Hamburg, Germany) using primers ACseqrev4120/ACseqfor3657 and probe 2 (644 bp) was synthesized using primers AC SeqIntRev2/ACM13ForRev (see Supplementary Table 1 for primer sequences).

To complement the mutation, the full length wild-type *acyA* was PCR-amplified from *E. festucae* Fl1genomic DNA using Platinum Pfx Polymerase (ThermoFisher Scientific) and primers AcyAF and AcyAR. The 8371 bp fragment comprising 859 bp upstream of the putative start site, 7170 bp of the open reading frame and 342 bp of the 3′ un-translated region was purified according to the manufacturer's instructions (DNA Clean and Concentrator -5, Zymo Research, Irvine, CA, USA) and protoplasts of Δ*acyA*34 and Δ*acyA*42 disruption mutants transformed as described above. Ectopic integration of wild-type *acyA* was confirmed by PCR using primers ACKOF/R (data not shown).

### Fungal growth on media supplemented with cAMP

Mycelial sections (approximately 1 mm^2^) were inoculated onto PDA plates supplemented with 2.5, 5.0 or 7.5 mM cAMP sodium salt (Sigma-Aldrich, St. Louis, MO, USA). Three control strains (*E. festucae* Fl1 wild-type plus two strains with ectopic insertions, *acyA*19 and *acyA*49*)* and three independent disruption mutants (Δ*acyA*34, Δ*acyA*42, and Δ*acyA*47) were inoculated in triplicate onto each medium supplemented with cAMP (including a no-cAMP control) in a randomized design. The radial measurement (mm) of the colony was taken at approximately 24 h intervals over 165 h. The first measurement was taken 20 h after the colonies were inoculated. The growth rate of each strain was obtained by least squares regression and the slope of the line used to represent the rate of radial growth in mm/h. Analysis of variance was used to estimate the effects of strain and cAMP concentration, and their interaction. Means for each strain and cAMP combination were obtained, together with the least significant difference (LSD—calculated at the 5% significance level) between means. The residual plot from the ANOVA was checked and displayed no evidence of heterogeneity of variance, thus the pooled LSD was used for comparing means at the same level of cAMP.

### Infection of *L. perenne* with *E. festucae* Fl1

Mycelial sections were inoculated into incisions created in the SAM of sterile 5 days old *L. perenne* seedlings growing on 1.5% (w/v) water agar, according to the method of Latch and Christensen (1985). Strains inoculated included the wild-type, mutants Δ*acyA*34, Δ*acyA*42 and Δ*acyA*47, plus two independent complementation strains, Δ*acyA*34*/acyA* and Δ*acyA*42*/acyA*. Inoculated plants were then maintained at 22°C in the dark for 7 days, followed by 22°C in the light for 7 days, before being transplanted into potting mix and maintained under glasshouse conditions. After 12 weeks, six tillers from each plant were tested for endophyte infection by tissue print immuno-assay using an *Epichloë*-specific polyclonal anti-serum (Simpson et al., 2012). Plants were inoculated as described on two independent occasions.

### Light microscopy

*E. festucae* Fl1 wild-type and *acyA* deletion mutants were grown in triplicate on potato dextrose broth (PDB, Difco, Le Pont, De Claix, France), either 1X or diluted 1:100 in water, containing 0.8% (w/v) agarose. The broths were inoculated and grown for 5 days at 22°C in continuous light. Alternatively, cultures were grown on the same medium on sterile microscope slides and incubated as described above. If grown in a culture dish, 0.5 cm^2^ of agar was cut from the outer edge of the colony, mounted onto a microscope slide, and a drop of water and a cover glass placed directly onto the specimen. If grown on a microscope slide, a drop of water was added to the mycelium and a cover glass applied. Cultures were imaged by bright field microscopy using a BX50 fluorescent microscope (Olympus, Tokyo, Japan) and a 40X UPLANFLN objective with a 0.75 numerical aperture. Images were taken using an Olympus Colorview III camera with AnalySIS^B^ image processing software. Endophytes in the epidermal layer of the host leaf sheath were stained with 0.15% (w/v) aniline blue and examined by bright field microscopy as described previously (Christensen et al., 2008). Leaf sheaths from two tillers per plant, and at least three infected plants per strain in each inoculation experiment, were examined.

### Confocal laser scanning microscopy (CLSM)

To examine hyphae in the host shoot apex, semi-thin (0.5–1 mm) longitudinal or transverse sections were taken through the shoot apex and true stem of at least six independent *L. perenne* tillers infected with each strain and mounted on a microscope slide in water. CLSM images were captured on an inverted FluoView FV10i Confocal Laser Scanning Microscope (Olympus). For visualizing EGFP the excitation wavelength was 457 nm and detection wavelength was between 465 and 565 nm. Two dimensional images were taken using the 10x phase contrast objective, numerical aperture 0.4 (equivalent to UPLSAPO 10x). To examine hyphal morphology in the expansion zone of the developing leaf, young leaves of approximately 5 cm in length were dissected from tillers and cut in half along the midrib. A 1 cm section from the base of a halved blade was mounted in water on a microscope slide for imaging as described above. For each image, the optimal depth to show the most hyphae possible in a sample was selected, and the laser sensitivity then adjusted to immediately below saturation levels.

### Transmission electron microscopy (TEM)

Endophyte-infected pseudostem material from two plants each infected with wild-type, Δ*acyA*34, Δ*acyA*42, or Δ*acyA*34/*acyA*, was dissected from *L. perenne* plants 0.5 cm above the tiller crown, and 0.5 mm transverse sections fixed in 2% (w/v) formaldehyde and 3% (w/v) glutaraldehyde in 0.1 M sodium/potassium phosphate (pH 7.2) for 2 h at room temperature. The samples were washed three times in 0.1 M sodium/potassium phosphate (pH 7.2) and post-fixed in 1% (w/v) osmium tetroxide in the same buffer for 1 h. After washing again as described above, the samples were subjected to a graded acetone/water series with 10 min each in 25, 50, 75, and 95% (v/v) acetone, followed by concentrated acetone for 2 h. The samples were embedded in Procure 812 resin (ProSciTech, Kirwan, Qld. Australia):acetone (50:50) overnight under constant stirring and then in 100% resin overnight. The resin was refreshed for a further 8 h and then mounted in 100% resin at 60°C for 48 h. Ultra-thin sections were collected onto a copper grid and stained with saturated uranyl acetate in 50% (v/v) ethanol for 4 min and then in lead citrate for a further 4 min. Specimens were examined at 46 000X magnification on a Philips CM10 (Philips Electron Optics, Eindhoven, The Netherlands) transmission electron microscope. Micrographs of at least 12 hyphae in developing leaves and in the second mature leaf sheath were captured. The thickness of hyphal cell walls was measured at eight equidistant positions around the circumference of each hypha using open source ImageJ 1.45 s software (Wayne Rasband, National Institutes of Health, USA). The cell wall thickness measurements were analyzed using one way ANOVA to compare the strains. The least significant differences (*P* = 0.05 level) between means were calculated.

### Detection of ROS

Superoxide radicals in *E. festucae* in axenic culture were stained using NBT (Sigma-Aldrich) as described by Tanaka et al. (2006). Three replicates for each strain were included in the analysis and the experiment was repeated twice. Strains were grown on PDA for 1 week at 22°C under 8 h light and 16 h dark, and mycelia stained for 5 h at 22°C in continuous light by incubation in 20 μL of 0.05% (w/v) NBT dissolved in 0.05 M sodium phosphate pH 7.5. The reaction was stopped by removing the stain and adding 40 μL of absolute ethanol. The ethanol was removed after 5 min, and mycelia were analyzed at 400X magnification under bright field illumination using an Olympus BX50 compound microscope with a 40X UPLANFLN objective and a 0.75 numerical aperture. Images were taken using an Olympus Colorview III camera with AnalySIS^B^ image processing software.

### Quantitation of hyphal biomass

Genomic DNA was extracted from freeze-dried pseudostems (leaf and blade tissue between the crown and the first ligule of a tiller) dissected from three tillers of each plant using the DNAeasy Plant Kit (Qiagen). The plants were infected with either the wild-type or Δ*acyA*42 mutant strain. There were three replicate plants per strain. Hyphal biomass (expressed as endophyte concentration) was determined by quantitative PCR of the single copy *E. festucae* NRPS-1 gene (EFM3.005350, http://www.endophyte.uky.edu/) from 1 ng of genomic DNA using a MyiQ™ cycler (Bio-Rad Laboratories, Hercules, California, USA), with primers 1-1F, and 1-1R (Supplementary Table 1) as described (Rasmussen et al., 2007; Liu et al., 2011). Hyphal biomass between strains was compared using one way ANOVA. The least significant differences (*P* = 0.05 level) between means was calculated.

## Results

### The *E. festucae acyA* gene

**Figure 1:**
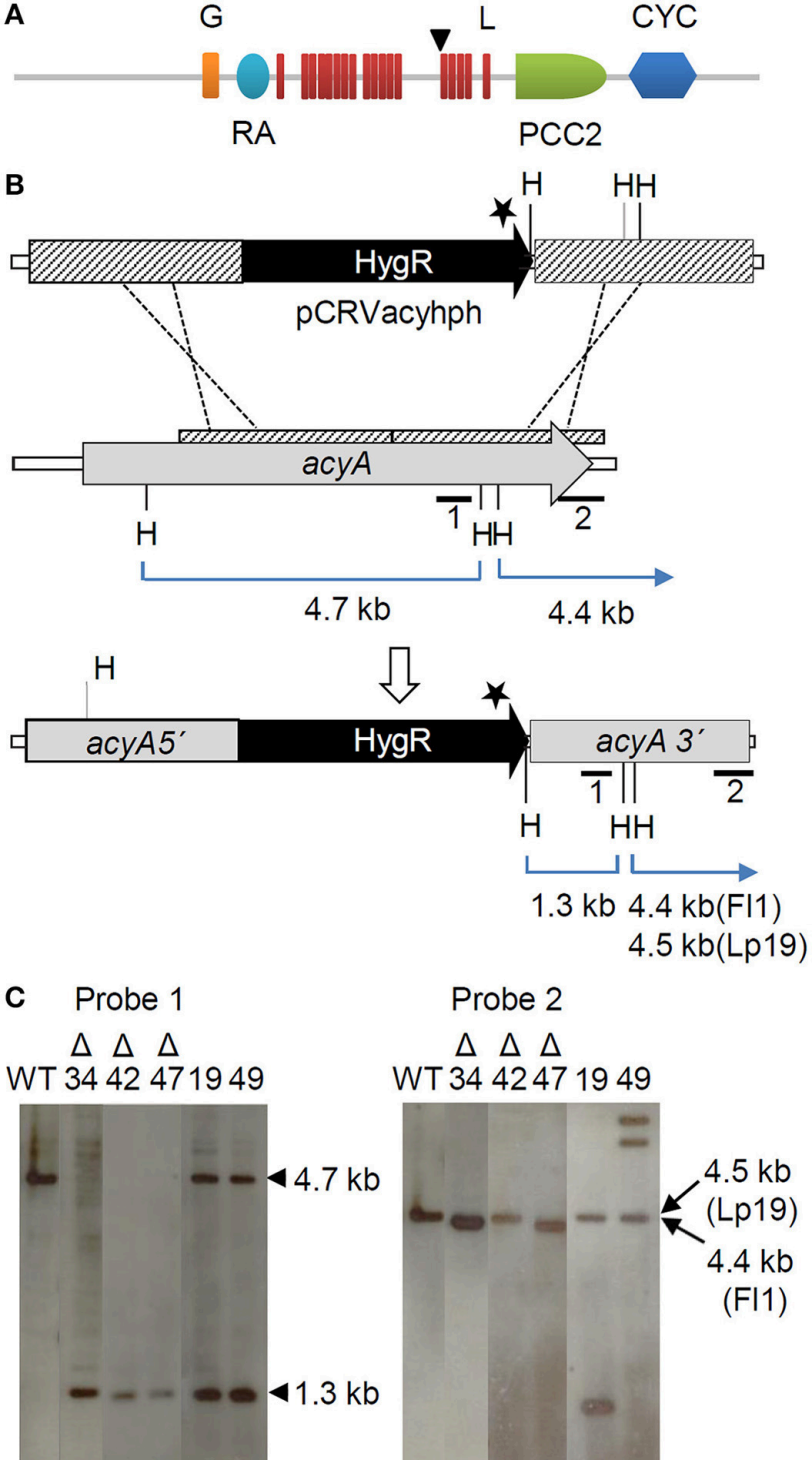
**Disruption of *acyA* in *E. festucae* Fl1. (A)** Domain architecture of AcyA in relation to the insertion site of the hygromycin resistance cassette (HygR). The protein contains the domains for G-alpha binding (G), ras association (RA), leucine rich repeats (L), protein phosphatase 2C (PP2C), and catalytic cyclic nucleotide biosynthesis (CYC) (Baker and Kelly, 2004). The HygR cassette was inserted (arrow) upstream of the phosphatase 2C (PPC2) and catalytic (CyC) domains. **(B)** Diagram of the *acyA* disruption locus in *E. festucae* Fl1. In vector pCRVacyhph, the HygR cassette is flanked by approximately 3 kb of DNA from the partial *acyA* gene (hatched) cloned from *E. festucae* var. *lolii* Lp19 (KR815911). The stop codon in the hygromycin resistance gene is indicated (⋆). The regions of homology between the Lp19 flanking regions and the corresponding Fl1 *acyA* sequence (hatched) is shown immediately above the Fl1 *acyA* gene, gene model Fl1M3.048730 (http://www.endophyte.uky.edu). Probes 1 and 2 (solid bars) were used in Southern-blot hybridization experiments **(C)** to confirm that the endogenous *acyA* gene had been disrupted and to ascertain the number of *acyA* copies in each strain respectively. **(C)** Southern-blot hybridization to confirm *acyA* insertion mutants. Genomic DNA was isolated from the wild-type (WT) plus a number of putative insertional mutants, and digested with *Hin*dIII. Probe 1 bound to the predicted 4.7 kb fragment in the wild-type *acyA* also in ectopic integrants *acyA*19 and *acyA*49 and to the 1.3 kb *Hin*dIII fragment of the disrupted *acyA* gene in ectopic integrants *acyA*19 & *acyA*49 and insertional mutants Δ*acyA*34 (Δ34), Δ*acyA*42 (Δ42) and Δ*acyA*47 (Δ47). Probe 2 was used to determine copy number, and bound to either a 4.4 kb or 4.5 kb *Hin*dIII fragment depending on whether the recombination locus was before or after the 66 bp indel in the Lp19 *acyA*.

A partial *acyA* gene (1419–5816 bp) was initially recovered from *E. festucae* var. *lolii* (Lp19). Subsequent to functional characterization of this gene in *E. festucae* Fl1 (reported here), the Fl1 genome became available and the full length *acyA* gene recovered (gene model Fl1M3.048730, http://www.endophyte.uky.edu/). Analysis of the partial and full length conceptual AcyA proteins from the Lp19 and Fl1 strains respectively, confirmed the presence of the key motifs consistent with fungal Class III adenylate cyclases, including the domains for adenylate cyclase G-alpha binding (IPR013716), ras association (IPR000159), leucine rich repeats (IPR001611, IPR003591, IPR025875), protein phosphatase 2C-like (IPR001932), and the adenylate cyclase (IPR001054) catalytic core (Figure 1A). The *E. festucae* var. *lolii acyA* sequence contains a 66 bp indel that is not present in the *E. festucae* Fl1 *acyA*. Consistent with other fungal species, Southern-blot hybridization confirmed that *E. festucae* Fl1 contains a single *acyA* gene (Figure 1C) and BLASTn analysis of the genomes of other *Epichloë* species (Schardl et al., 2013) also suggests the presence of a single *acyA* gene in the haploid strains examined (Table 2).

Since genome hybridization has been a relatively common occurrence in the *Epichloë* genus, we investigated whether two recently-sequenced allopolyploid strains had retained both copies of the *acyA* gene from their progenitors, with the attendant prospects for neofunctionalisation (as is observed in mammals). Reads from the genomes of strains AR1006 (*E. uncinata*) and AR3046 (*Epichloë* sp.) were mapped independently to the haploid genomes of extant strains related to the original progenitors. *E. uncinata* (AR1006) is a hybrid between *E. bromicola* and *E. typhina* (Craven et al., 2001; Moon et al., 2004), and AR3046 is a hybrid between *E. bromicola* and the *E. baconii*/*E. amarillans* clade (unpublished). Two *acyA* genes were recovered from each allopolyploid genome examined (Table 2), consistent with a gene originating from each of the species that contributed to the allopolyploid. The *acyA* homeologs from AR3046 (KT732649 and KT732650) and AR1006 (KT732647 and KT732648) were then mapped back to the genomes of the predicted progenitor species, which confirmed that each *acyA* gene mapped with higher identity to one or the other of the species that contributed the genes to the allopolyploid (Supplementary Table 2A). The *acyA* genes from AR3046 encoded conceptual full-length proteins indicating that both genes had the potential to be functional, however in AR1006 one of the genes (KT732647) encoded a full length protein while the other, KT732648, encoded a conceptual protein truncated after amino acid position 686. This gene has also lost a triplet 232–234 bp from the start codon and sustained a number of other deletions relative to the presumed parent *E. typhina* (Supplementary Table 2B). Alignment of *acyA* genes within each allopolyploid strain indicated they had no more identity between each other than was found in the comparisons between genes from other species (Supplementary Table 2C) confirming that the genes came together through a genome hybridization event and was not the result of gene duplication.

### Disruption of *E. festucae* Fl1 *acyA*

An *acyA* gene disruption vector (pCRVacyhph) was constructed using flanking regions designed from the partial *E. festucae* var. *lolii* Lp19 sequence, and gene disruption experiments were performed in strain Fl1 as the frequency of homologous recombination in Lp19 is extremely low. Insertion of the hygromycin cassette introduced a stop codon into the *acyA* open reading frame upstream of the catalytic core domain (Figures 1A,B). Three colonies (Δ*acyA*34, Δ*acyA*42, and Δ*acyA*47), each with a single gene disruption event and no ectopic insertions, were identified by PCR and confirmed by Southern-blot hybridization (Figure 1C). The recombination breakpoints of the mutant strains differed with respect to the presence or absence of the 66 bp indel (Figure 1C), presumably due to differences in the recombination site during homologous recombination in the disruption mutants. *E. festucae* Δ*acyA*34 contained the insertion, while Δ*acyA*42 and Δ*acyA*47 did not. The integrity of each gene immediately flanking the disrupted *acyA* was checked by PCR to ensure that the disruption event was confined to the *acyA* gene. Two primer pairs spanning the intergenic region and the flanking gene 5′ and 3′ of the *acyA* were used. No evidence of untargeted rearrangements were detected (Supplementary Figure 1). Two further colonies (*acyA*19 and *acyA*49) retained the intact *acyA* gene plus an ectopic insertion of the gene replacement vector and were used as transformation controls.

### Regulation of radial growth by cAMP signaling in *E. festucae* in axenic culture

**Figure 2:**
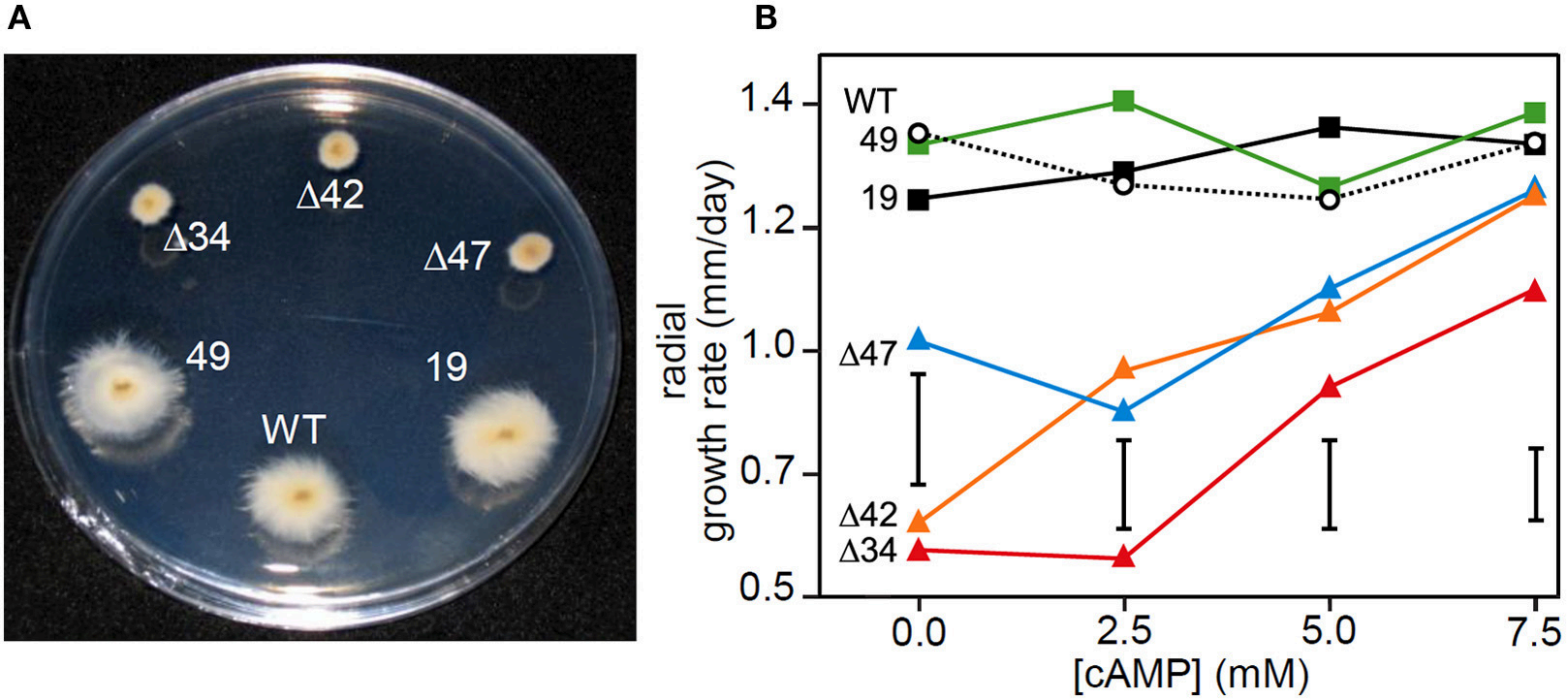
**Growth of *E. festucae acyA* disruption mutants in axenic culture**. **(A)** Growth of wild-type, disruption mutants Δ*acyA*34 (Δ34), Δ*acyA*42 (Δ42), ΔacyA47 (Δ47), and colonies with an intact *acyA* gene plus an ectopic insertion(s) of the transformation vector, *acyA*19, *acyA*49 on PDA (22°C for 7 days). **(B)** The effect of exogenous cAMP on the radial growth rate of the same strains. The mean growth rate of each strain (with three clonal replicates) at each cAMP concentration is shown. Analysis of variance was used to compare the growth rate of strains between and within each concentration of cAMP. The vertical bar represents the least significant differences (LSD) between means at the 5% significance level when comparing strains at the same concentration of cAMP.

The three independent *E. festucae* mutants, Δ*acyA*42, Δ*acyA*34, and Δ*acyA*47, grew more slowly in axenic culture compared with the wild-type, or control strains *acyA*19 and *acyA*49 (Figure 2A). The mutant colonies were also highly compact compared to the controls. The radial growth rates of Δ*acyA*42, Δ*acyA*34, and Δ*acyA*47 increased in a dose-dependent manner in response to supplementation of the media with exogenous cAMP (Figure 2B). The growth rates of *E. festucae* Δ*acyA*42 and Δ*acyA*47 were statistically indistinguishable from wild-type when the medium was supplemented with 7.5 mM cAMP. *E. festucae* Δ*acyA*34 grew more slowly than the other strains under all conditions; however its growth rate on PDA containing 7.5 mM cAMP was almost 2 fold higher than when growing on PDA alone. The growth rates of the control strains (wild-type, *acyA*19 and *acyA*49) were not altered by cAMP supplementation (Figure 2B) indicating that endogenous cAMP does not limit growth of strains with functional AC enzymes.

*E. festucae* Δ*acyA*47 produced a faster-growing sector (named Δ*acyA*47var) on PDA supplemented with 150 μg/mL hygromycin. Southern-blot hybridization confirmed that the sector was identical to Δ*acyA*47 at the disruption locus (data not shown). Strains Δ*acyA*34 and Δ*acyA*42 did not produce overt spontaneous growth revertants, however if repeatedly sub-cultured onto fresh media, gradually grew faster until their growth rates were similar to wild-type (data not shown). PCR (using primers ACKO F and ACKO R) was used to check the fidelity of the integration locus during this period (data not shown). We saw no evidence of a loss of the integrated DNA over time in these colonies suggesting that the changes in phenotype were due to mutations or epigenetic changes at other loci.

### Regulation of hyphal branching in *E. festucae* growing in axenic culture

**Figure 3:**
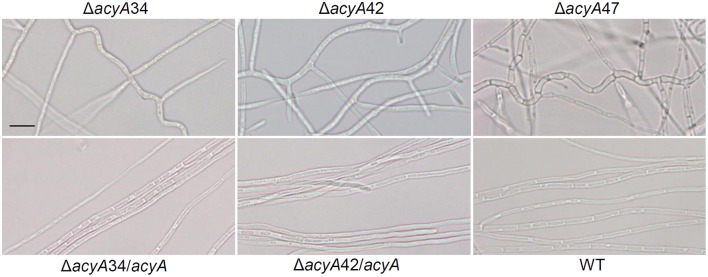
**Effects of *acyA* disruption on *E. festucae* hyphal morphology in axenic culture**. Bright field images of *E. festucae* Fl1 hyphae growing on water agar at 22°C for 7 days (bar, 10 μM). Images were taken approximately 3 mm behind the colony margin. Hyphae of all independent mutants (Δ*acyA*34, Δ*acyA*42, and Δ*acyA*47) were convoluted, irregular in diameter (Δ*acyA*47) and heavily branched compared to the wild-type. Ectopic integration of the wild-type *acyA* gene into Δ*acyA*34 and Δ*acyA*42 resulted in a reversion to the wild-type phenotype to greater or lesser extents dependent on the individual strain (Δ*acyA*34/*acyA*, Δ*acyA*42/*acyA*). Gross morphology at the hyphal tips appeared similar in mutants and wild-type.

We next examined the hyphal morphology of *acyA* disruption mutants in culture by bright field microscopy. In wild-type cultures, hyphae were long and straight, relatively sparsely branched and produced lateral branches at the proximal end of compartments immediately adjacent to septa several compartments behind the tip (Figure 3). Conversely, the hyphae of *E. festucae* Δ*acyA*34, Δ*acyA*42, and Δ*acyA*47 were highly convoluted, sometimes forming thick aggregates or cables, and produced multiple lateral branches. Excessive lateral branches accounted for the compact nature of the mutant colonies. The morphology of strains complemented with the wild-type *acyA* gene were similar to the wild-type strain (Figure 3) confirming the role of cAMP in suppressing lateral branches. This phenotype was reproducible, however similarly to the observations on effects of AC disruption on colony growth rate, hyphal morphology in mutant colonies was not stable over time and reverted to the wild-type form if the cultures were maintained continuously in axenic culture.

### Regulation of ROS in *E. festucae* growing in axenic culture

**Figure 4:**
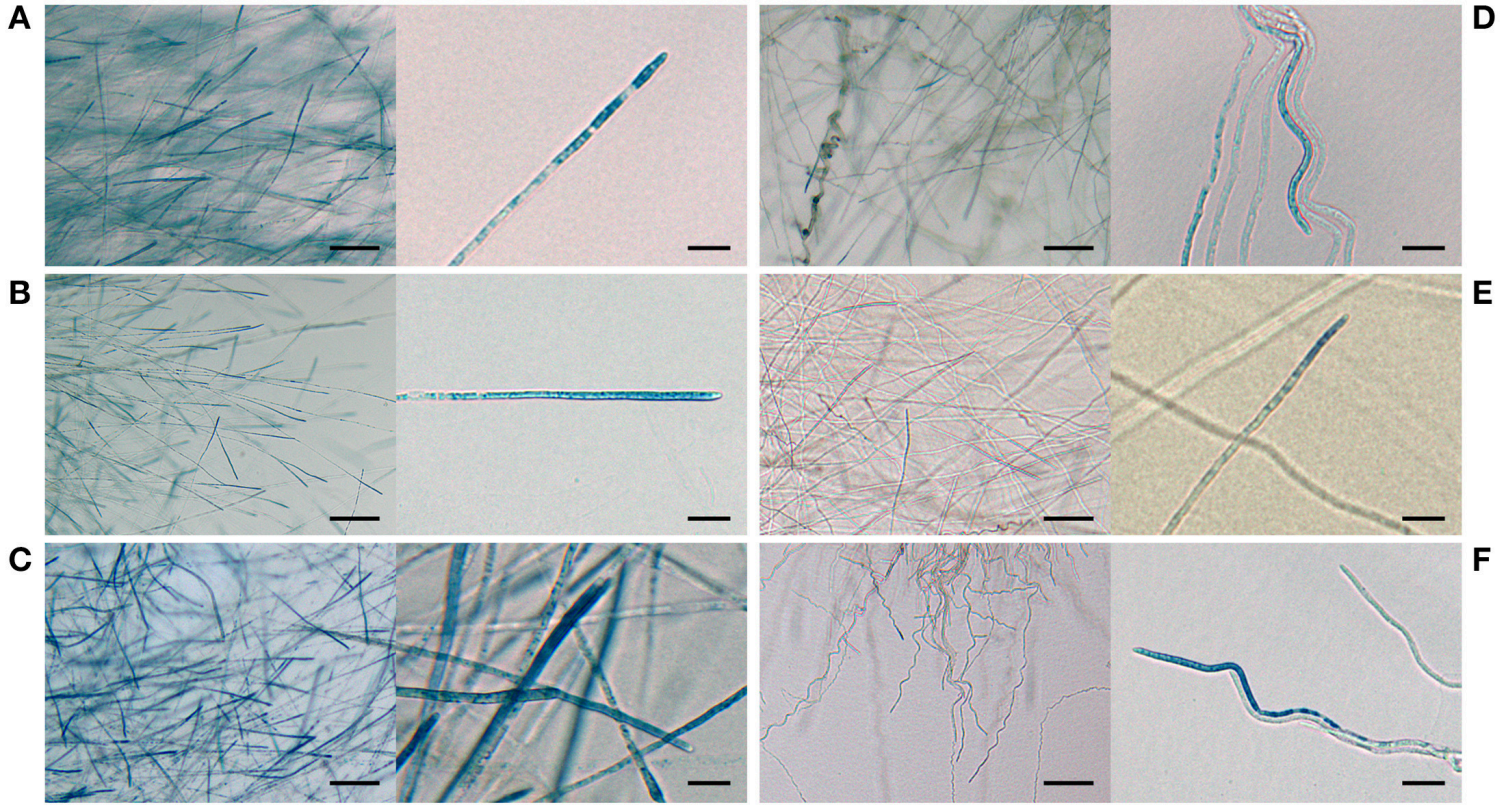
**Influence of *acyA* disruption on production of ROS in *E. festucae* Fl1 hyphae in axenic culture**. Bright field images of *E. festucae* Fl1 growing on PDA at 22°C for 7 days and stained with NBT. Included is a low (scale bar = 50 μm) and high (scale bar = 5 μm) resolution image of the wild-type **(A)** plus mutants Δ*acyA*34 **(D)**, Δ42 **(E)**, and Δ47 **(F)**, and complementation strains Δ*acyA*34/*acyA* **(B)** and Δ*acyA*42/*acyA* **(C)**.

Synthesis of ROS is essential for mutualism in the *E. festucae* Fl1/*L. perenne* interaction, and disruption results in hyper-colonization of host tissues, stunting and premature leaf senescence (Tanaka et al., 2006, 2008). In order to determine whether the morphology of the *E. festucae* Δ*acyA* disruption mutants was linked to changes in ROS production, strains were grown in culture on microscope slides and stained with NBT. NBT forms a blue precipitate on exposure to superoxide ions, and blue deposits were typically observed in the hyphal apices of the *E. festucae* Fl1 wild-type (Figure 4) and occasionally in some compartments behind the tip (data not shown). NBT staining of *E. festucae acyA* disruption mutants revealed a substantial reduction in superoxide radicals relative to the wild-type strain (Figure 4). The localization of superoxide radicals was similar between the wild-type and mutants, however superoxide ions were not detectable in the majority of mutant hyphae (Figure 4). Strains complemented with the wild-type *acyA* gene were able to produce ROS at similar or higher levels than the wild-type confirming that cAMP signaling directly or indirectly regulates accumulation of superoxide ions in *E. festucae*.

### Regulation of *E. festucae* growth by cAMP during colonization of *L. perenne*

To determine the role of fungal cAMP signaling in *E. festucae* during mutualistic interactions with *L. perenne*, mycelia of strains carrying the disrupted gene were inoculated though a small incision into young seedlings. In each inoculation experiment, between 25 and 50% of inoculated plants (*n* = 25) became infected with wild-type, Δ*acyA*34, Δ*acyA*42, and the complementation strains, however despite repeated attempts (*n* > 75 plants), no plants infected with the original *E. festucae* Δ*acyA*47 strain were obtained. It is unclear why this strain was apparently incapable of host colonization since the *acyA* locus was identical to Δ*acyA*34 and Δ*acyA*42. Inoculation of plants with *E. festucae* Δ*acyA*47var produced infected plants with similar frequency to the other strains. This strain was not included in further experiments due to its unstable phenotype.

**Figure 5:**
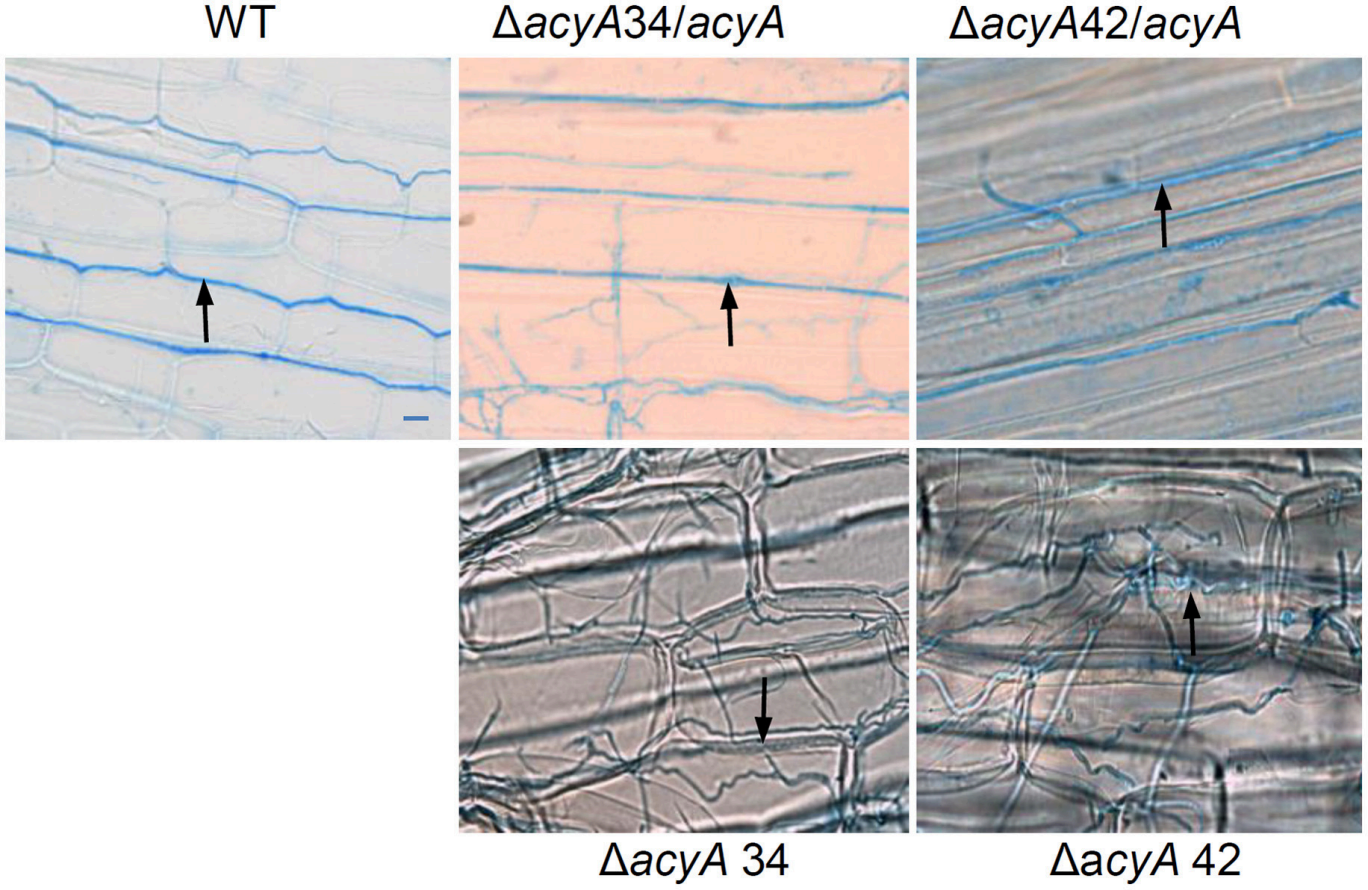
**Morphology of *E. festucae* Δ*acyA* in *L. perenne.*** Aniline blue-stained hyphae (arrows) in the mature second leaf sheath of *L. perenne* infected with the wild-type and strains Δ*acyA*34 and Δ*acyA*42. The morphology of the mutant strains after complementation (Δ*acyA*34/*acyA* and Δ*acyA*42/*acyA*) with the wild-type gene is also shown. All images are at the same magnification. The scale bar is 5 μm.

**Figure 6:**
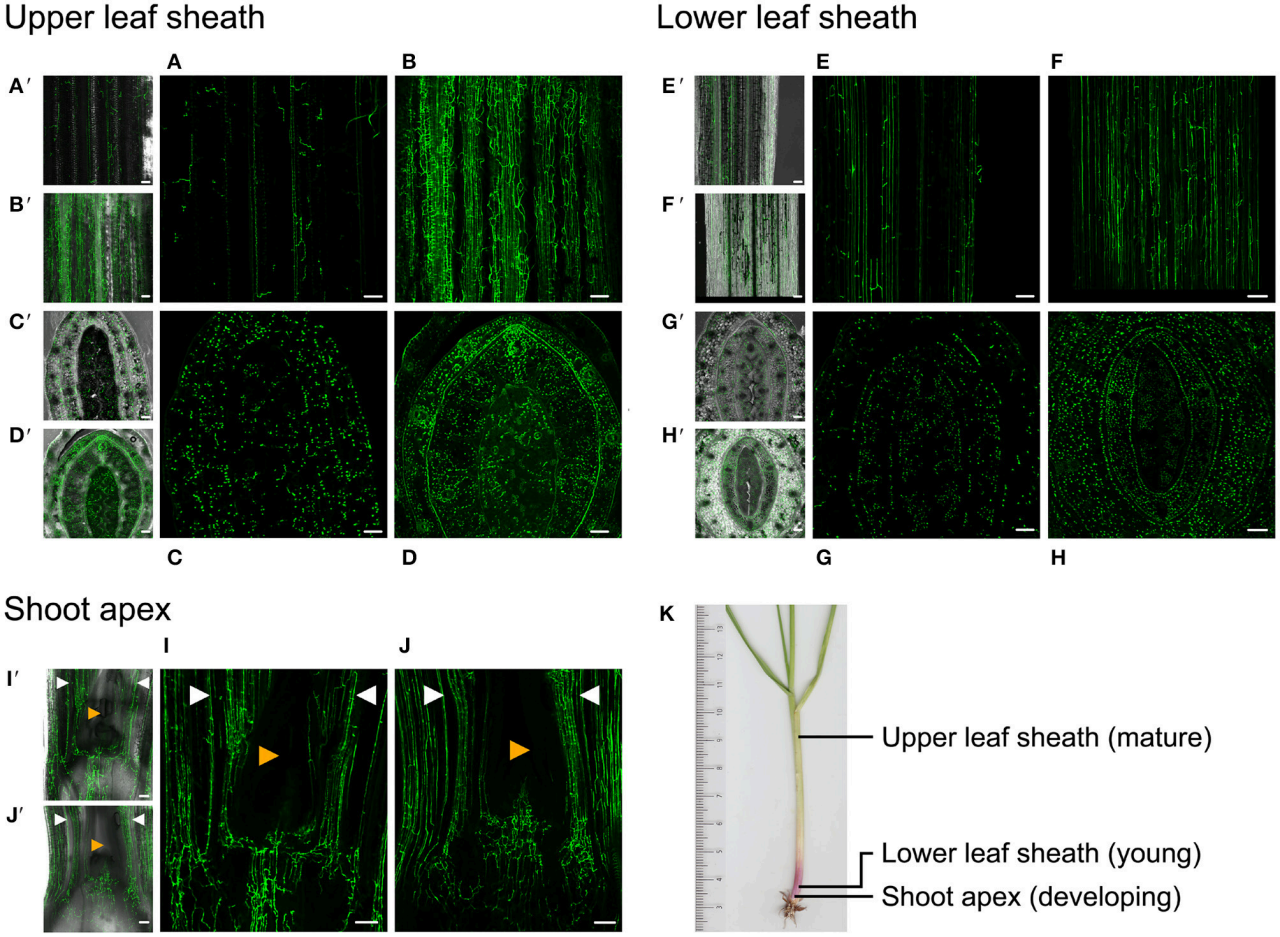
**Disruption of *E. festucae acyA* deregulates hyphal branching during host colonization**. **(A–J)**. Confocal laser scanning micrographs (CLSM) of *E. festucae* hyphae at three stages of host development. *L. perenne* leaves infected with the wild-type and disruption mutant Δ*acyA*42 are shown. Both strains are transformed with plasmid pTEFEGFP and constitutively express EGFP. **(A**′**–J**′**)**, CSLM overlaid on corresponding phase contrast images of the same field of view. **(K)** *L. perenne* tiller showing the positions at which the tissue sections were taken. A/B, CLSM of a longitudinal section of the mature upper leaf sheath of the pseudostem 3.5 cm above the crown (base) of the tiller showing colonization by WT and Δ*acyA*42 hyphae respectively. **(C,D)** CLSM of a transverse section though the tiller at the same position as **(A,B)**. The youngest developing leaf is in the center of the section and is enclosed by progressively older leaf sheaths. **(E,F)** CLSM of a longitudinal section of a young developing leaf sheath immediately above the crown of the tiller. (**G,H)** CLSM of a transverse section through the pseudostem at the same position as (**E,F)**. **(I,J)** CSLM of a longitudinal section through the crown showing the position of hyphae relative to the shoot apex. The shoot apex (red arrow) is a small dome of overlapping leaf primordia visible only in the phase contract images **(I**′**, J**′**)**. Hyphae are either not detectable in this tissue by confocal microscopy or are not present. Hyphae in the youngest leaves emerging from the shoot apex are marked by white arrows.

**Figure 7:**
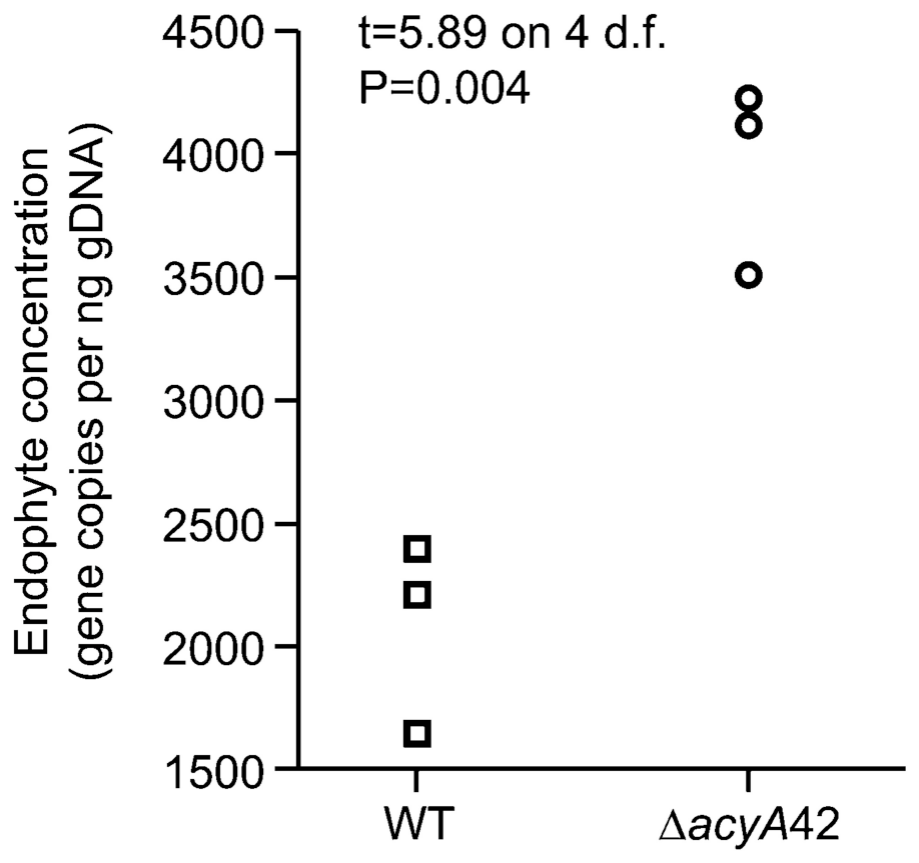
**Effects of *acyA* disruption on biomass of *E. festucae* in host grasses**. Comparison of hyphal biomass in the pseudostem (the region between the crown and the first ligule of a tiller) of plants infected with either the wild-type or Δ*acyA*42 mutant strains. Hyphal biomass (expressed as endophyte concentration) was determined by quantitative PCR of a single copy gene from 1 ng of genomic DNA. Hyphal biomass between strains was compared using the Students' *T*-test. The means of the two treatments were significantly different (*P* = 0.004).

We next investigated whether a functional fungal cAMP signaling pathway is required for a mutualistic interaction between the symbionts and the host grass. Three to five independent plants for each strain were examined. Consistent differences in the growth and phenotype of plants infected with wild-type, Δ*acyA*, or Δ*acy/acyA* complemented strains were not observed, although plants infected with mutant strains did express a variable marginally-stunted phenotype (Supplementary Figure 2). After 3 months, hyphae within the mature leaf sheaths of infected plants were stained with aniline blue and analyzed by bright field microscopy. Wild-type hyphae in this tissue were long and straight, seldom branched, and oriented in the direction of leaf growth. In contrast, hyphae of the two independent *acyA* mutants were highly branched and convoluted (Figure 5). The phenotypes of Δ*acyA*34 and 42 in all the plants examined (at least three plants per strain per experiment) were highly consistent. The complementation strains Δ*acyA*34*/acyA* and Δ*acyA*42*/acyA* both resembled the wild type (Figure 5). To further investigate the role of cAMP signaling in endophyte colonization of the host at different developmental stages, we transformed Δ*acyA*42 with vector pTEFEGFP (for constitutive EGFP expression in *E. festucae*) and inoculated EGFP-expressing strains into *L. perenne* seedlings for examination by confocal microscopy. Wild-type *E. festucae* Fl1 transformed with the same plasmid was used as a control. An EGFP-expressing *acyA*-complemented strain was not included in this experiment as it was not technically feasible to conduct a third transformation on this mutant strain. We first examined the hyphae in longitudinal sections taken through the shoot apex at the base of the tiller. In host tissues immediately below the meristem, and in the youngest developing leaves, the mycelial density of Δ*acyA*42/EGFP was similar to wild-type controls (Figures 6I,J), however in young leaf sheaths above the shoot meristem (lower leaf sheath), hyphae of the mutant strain appeared more numerous than wild-type (Figures 6E–H). A key feature of *E. festucae*-*L. perenne* mutualism is that once host tissues are mature and have stopped expanding, colonizing endophytes also cease growing (Tan et al., 2001). To determine whether *E. festucae* Δ*acyA*42/EGFP was still capable of responding to host developmental cues, and to stop growing, epidermal peels from mature (upper) leaf sheaths were examined. Contrary to the uniform appearance of wild-type hyphae in this tissue, disruption of the cAMP signaling pathway resulted in a dense and heavily-branched mycelium (Figures 6A–D). To confirm the microscopy observations, the biomass of the wild-type and Δ*acyA*42 strains in the pseudostem of these plants was determined by quantitative PCR of a single copy *E. festucae* gene. Hyphal biomass (expressed as endophyte concentration) was an average of 2081 (SE 222.7) gene copies per ng of plant and endophyte genomic DNA for wild-type, and 3949 (SE 226.1) for the Δ*acyA*42 mutant, 1.9 fold higher than wild-type (Figure 7). The means of the technical replicates for each biological replicate per strain were compared using the Student's *T*-test. The *T*-test indicated that the mean hyphal biomass of the mutant strain was significantly greater than the wild-type (*P* = 0.004).

**Figure 8:**
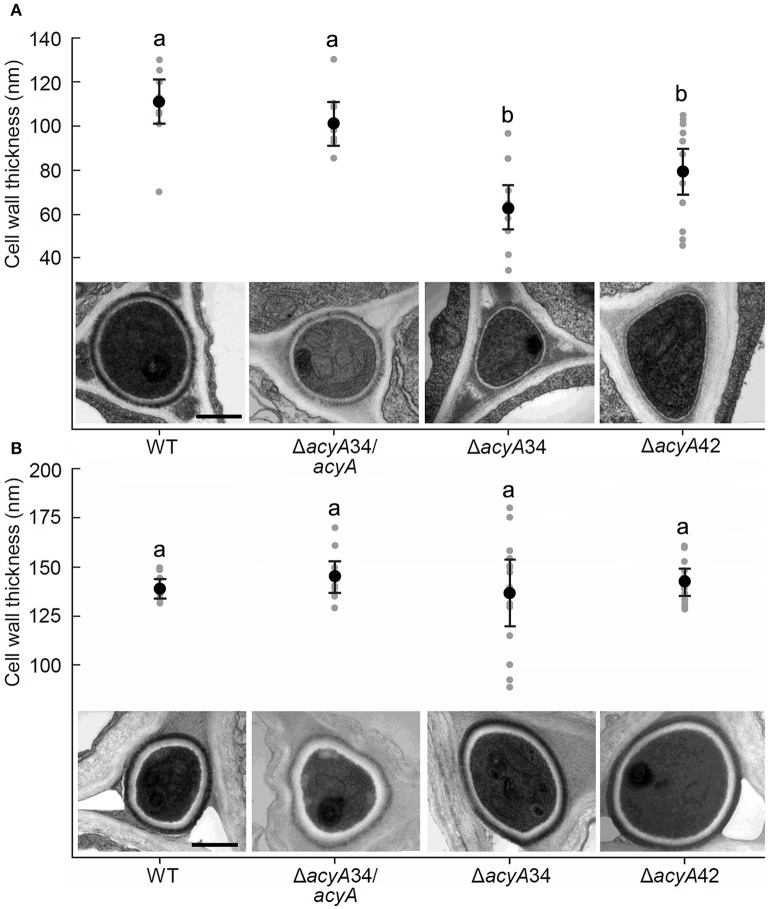
**Regulation of hyphal cell wall synthesis by cAMP during leaf colonization**. The cell wall of 12 hyphae each from developing leaves **(A)** and mature second leaf sheaths **(B)** of *L. perenne* was measured at eight positions around each hypha. The gray symbols represent the average cell wall thickness of each hypha. The overall mean cell wall thickness and its 95% confidence interval is presented for each strain. Analysis of variance was used to compare the strains. Within each plant tissue **(A,B)**, different letters indicate means that are different at the 5% significance level. Transmission electron micrographs show representative hyphae of *E. festucae* wild-type, mutants Δ*acyA*34, and Δ*acyA*42, and the complementation strain Δ*acyA*34/*acyA* strain in the two host tissue types. All images are at the same magnification. The scale bar is 500 nm.

The impact of *acyA* disruption on hyphal ultrastructure *in planta* was investigated by TEM of infected *L. perenne* tillers. Tillers are comprised of bundles of leaves ranging in development from the most immature (in the middle of the tiller) to the fully mature outer leaf sheath. Transverse sections through the base of tillers were fixed and embedded. Ultra-thin sections of tillers infected with wild-type, Δ*acyA*34, Δ*acyA*42, and the Δ*acyA*34*/acyA* complementation strains were stained with osmium tetroxide and examined by TEM. The thickness of the cell wall was measured at eight equidistant positions around 12 hyphae from the immature leaf blade (Figure 8A) and the second fully mature leaf sheath (Figure 8B) of each tiller. In immature leaves, where both the plant and endophyte grow by intercalary growth, mean cell wall thickness of Δ*acyA*34 and Δ*acyA*42 was 70.3 and 62.8 nm respectively, significantly thinner (*P* ≤ 0.05) than the wild-type which had an average thickness of 111.3 nm (Figure 8A). The phenotype was nearly fully restored in the Δ*acyA*34*/acyA* complemented strain which had a mean cell wall thickness of 101.04 nm, indicating that cAMP signaling positively regulates cell wall biogenesis during intercalary growth. Conversely, in the fully developed leaf sheath where plant and fungal cells are no longer growing, there were no significant differences in cell wall thickness between any of the strains (Figure 8B). Cell walls of wild-type *E. festucae* hyphae in the leaf sheath were thicker than those in the immature leaf (139 nm vs. 111.3 nm respectively, *P* ≤ 0.001) confirming previous reports of thinner cell walls in *E. festucae* Fl1 hyphae in developing vs. mature host tissues (Christensen et al., 2008).

## Discussion

In this study we tested the hypothesis that cAMP signaling is required for regulated growth of the mutualistic fungal endophyte *E. festucae* in host grasses, and for compatibility between the host and endophyte. Our data show that disruption of the *acyA* gene severely reduced *E. festucae* radial colony growth in axenic culture, however this growth defect did not affect initial plant infection processes. The *acyA* mutant strains were hyper-branched in culture and unable to accumulate superoxide radicals. When infecting plants, the mycelia became progressively more dense compared to wild-type as plant tissues aged. Disruption of cAMP synthesis therefore disrupted the ability of the endophyte to grow in synchrony with developing leaves, an attribute which prevents overgrowth of the host during symbiosis.

The reduction in colony radial growth rate of *E. festucae acyA* mutants is consistent with deletion of the AC gene in plant pathogenic fungi such as *F. verticillioides* (Choi and Xu, 2010), *F. fujikuroi* (García-Martínez et al., 2012), *F. proliferatum* (Kohut et al., 2010) and *Botrytis cinerea* (Klimpel et al., 2002). Complementation of the mutant phenotype with cAMP confirmed that the relatively slow radial growth rate in the mutants was due to cAMP depletion. Significantly, strains Δ*acyA3*4 and Δ*acyA*42 had similar radial growth rates in the absence of exogenous cAMP, while Δ*acyA*47 grew at a faster rate despite having an identical *acyA* disruption locus. Similar spontaneous revertants have been obtained from adenylate cyclase *MAC1* mutants of *Magnaporthe oryzae* (Adachi and Hamer, 1998) and *cr-1* mutants of *Neurospora crassa* (Garnjobst and Tatum, 1970). This phenomenon has also been reported in *B. cinerea* where the slow radial growth rate of the AC mutants reverted to wild-type levels over time (Klimpel et al., 2002). The reason for this phenomenon was not investigated in our study but is speculated to be due to the accumulation of suppressor mutations that complement the mutant growth phenotype. By-pass suppressors of the *MAC1* (AC) phenotype (*sum*) that fully restore growth and morphogenesis in *M. oryzae* have been identified (Adachi and Hamer, 1998). One such mutation in the PKA catalytic subunit (*sum1–99*) alters a conserved amino acid in the cAMP binding domain (Adachi and Hamer, 1998). As shown here and reported elsewhere, the fungal cAMP signaling pathway is highly responsive to AC deletion/disruption and perturbations in cAMP synthesis. Suppressor mutations mask AC deletion phenotypes and confound the interpretation of cAMP signaling experiments, potentially contributing to the diversity in morphology and growth rates reported in different fungi.

Microscopic examination of *E. festucae* Δ*acyA* strains in culture revealed that the cAMP signaling pathway plays a significant role in colony architecture. Mutant hyphae were aggregated, convoluted and hyper-branched. The cAMP cascade therefore enforces apical dominance in this species by preventing development of new hyphal branch points near the apex. The morphology of the *acyA* mutant colonies quite closely resembled the *E. festucae* small GTPase *racA* mutant (Tanaka et al., 2008) which also had a slower growth rate compared to the wild-type, had convoluted hyphae, produced lateral branches with higher frequency, and formed branches at atypical sites. Notably, similarly to the *E. festucae* Δ*acyA* phenotype, hyphal morphology and colony size in the Δ*racA* reverted to wild-type over time in culture (Kayano et al., 2013). RacA is a small GTPase of the Rho subfamily involved in hyphal growth and morphogenesis (Zhang et al., 2013). Production of superoxide from molecular oxygen by the plasma membrane-localized NADPH oxidase complex is important in regulating polar growth in *E. festucae* as exemplified by deletion of several genes encoding proteins of the NADPH oxidase complex (Takemoto et al., 2007; Scott and Eaton, 2008; Eaton et al., 2011; Tanaka et al., 2012), *noxA* (Tanaka et al., 2006), *noxR* (Takemoto et al., 2006), *racA* (Tanaka et al., 2008), and *bemA* (Takemoto et al., 2011). *E. festucae* RacA GTPase binds to NoxR, a regulator of NADPH oxidase, and activates the NADPH oxidase complex that synthesizes superoxide (Tanaka et al., 2008). A further similarity in morphology between the *E. festucae racA* and *acyA* mutants in culture was the absence of ROS in hyphal tips. In the strains complemented with the wild-type *acyA* gene, superoxide levels were the same as, or more concentrated than wild-type, confirming that cAMP signaling positively regulates superoxide accumulation in *E. festucae*. This result contrasts with the *E. festucae* Fl1 *sakA* mutant where deletion of the stress-activated mitogen-activated protein (MAP) kinase resulted in elevated H_2_O_2_, both in culture and in plants (Eaton et al., 2008, 2010) suggesting the stress-activated MAP kinase and cAMP pathways play opposing roles in ROS regulation. Transcriptomics analysis of the *sakA* mutant demonstrated that genes for 27 ROS decomposition enzymes such as peroxidases and catalases are also more highly expressed in relation to the wild-type, possibly in response to the elevated ROS produced by this mutant (Eaton et al., 2010). Regulation of ROS by cAMP has also been reported in *C. albicans* where cAMP negatively regulates oxidative stress response genes such as *MCR1* (cytochrome *b*_5_ reductase), *SOD2* (Mn superoxide dismutase), *HSP12* (heat shock protein) and *CCP1* (cytochrome *c* peroxidase) (Bahn et al., 2007). Likewise, repression of the Ras-cAMP-PKA cascade by farnesol (a small signaling molecule) results in up-regulation of catalase and superoxide dismutase, with a consequential increase in resistance against oxidative stress (Deveau et al., 2010). Increased resistance to H_2_O_2_ has also been observed in *F. proliferatum* after deletion of *FpacyA* (Kohut et al., 2010). Despite similarities between *racA* and *acyA* mutants in morphology and ROS regulation during saprotrophic growth, deletion of *noxA, noxA/noxB, noxR*, or *bemA* in *E. festucae* results in only a slight reduction in colony growth on PDA and no marked effects on hyphal morphology (Tanaka et al., 2006; Kayano et al., 2013) indicating that the substantial reduction in radial colony growth rate and the hyper-branched phenotype of the *acyA* mutants on PDA is therefore not mediated through ROS but through other, yet unidentified, mechanisms. This is also true for traits associated with apical dominance in other fungi where apical dominance and polar growth is not overcome by disruption of NADPH oxidase genes when growing on enriched media (Scott and Eaton, 2008; Semighini and Harris, 2008). In *E. festucae noxA, noxA/noxB*, and *noxR* (but not *bemA*) are critical for regulation of apical dominance and hyphal organization during growth on water agar suggesting that ROS regulation of hyphal branching is more important in nutrient limited conditions (Kayano et al., 2013).

The cAMP signaling cascade regulates key processes in fungal pathogenesis, and in most pathogenic fungi AC deletion attenuates or eliminates virulence in animal and plant pathogens (Choi and Dean, 1997; Klimpel et al., 2002; Kohut et al., 2010), although others report the virulence unchanged, as in *F. fujikuroi* (Δ*acyA*) on tomato (García-Martínez et al., 2012). In contrast with most fungal pathogens, AC was not required for establishment of a stable symbiosis between *E. festucae* and *L. perenne*. Only Δ*acyA*34 and 42 were competent to form a symbiosis, and Δ*acyA*47 did not appear to be infectious, despite growing more rapidly in culture as mentioned above. The spontaneous growth revertant, Δ*acyA*47var, was capable of forming compatible interactions with host plants. We speculate that this change in infectivity was also due to suppressor mutation(s). The hyphae of *acyA* mutants in plants were hyper-branched in mature host tissues, suggesting that the endophytes produced many more hyphal apices in plants, as they did in culture. This phenotype largely reverted to wild-type in strains complemented with the functional *acyA* gene. *E. festucae* is predominantly a foliar symbiont, and infects aerial plant tissues as they are developing on the host SAM at the base of the plant. The youngest host (and endophyte) tissues are those nearest the SAM while the older tissues are those furthest from it (the tips of the leaves for example). Hyphae were visible between meristematic cells at the base of developing leaves, and morphology and distribution of Δ*acyA*42/EGFP appeared similar to the wild-type in this very young tissue. However hyphal biomass appeared to increase at each successive stage in leaf development examined. This contrasts with the wild-type colonization process where branching is mostly confined to hyphae colonizing meristematic host tissues, and is quite tightly constrained during intercalary growth in the host expansion zone (Christensen et al., 2008).

It is not possible to deduce whether breakdown in regulated control of hyphal biomass during host colonization through cAMP disruption was a consequence of lower ROS levels as this was not tested directly. However, *E. festucae* strains with deletions in components of the NADPH oxidase complex exhibit similar hyper-colonization phenotypes (Takemoto et al., 2006, 2011; Tanaka et al., 2006, 2008) suggesting that changes in ROS accumulation should be considered as one potential contributor to the *E. festucae* cAMP disruption phenotype in plants. Another *E. festucae* Fl1 mutant (Δ*sidN*, deficient in the biosynthesis of the iron-chelating siderophore epichloënin A), also phenocopies the defective polarized growth of the *E. festucae racA* and *acyA* deletion mutants, but under iron depleted conditions (Johnson et al., 2013b). Deletion of Δ*sidN* in *E. festucae* reduced *racA* transcripts between 2.2 and 3.3 fold in infected plants and the mutants also over colonized the host (Johnson et al., 2013b). Iron homeostatic regulation of the NADPH oxidase complex is therefore another layer of complexity potentially involved in ROS regulation of hyphal branching. Whether there are direct interactions between the NADPH oxidase complex, *racA*, iron homeostasis and the cAMP pathway remains to be determined. Unlike the other *acyA* phenotypes which revert to wild-type over time in culture, ROS suppression in *acyA* deletion mutants was highly stable. Cyclic AMP may therefore exert its effect on ROS accumulation through a different downstream mechanism to those used to regulate other traits such as colony growth and hyphal morphology.

The hyphal walls of the *E. festucae* Δ*acyA* mutants were significantly thinner compared to wild-type or *acyA*-complemented mutant strains, however this was only the case for hyphae in developing host tissues (where the endophytes grow by intercalary extension). This suggests that, while cell wall growth is slower in the *acyA* mutants vs. the wild-type, the differential is sufficiently small to enable them to maintain cell walls of similar thickness once hyphal and plant growth has ceased (in mature leaves). These data indicate that, similar to the wild-type, cell wall synthesis in *acyA* mutant strains also continues after extension growth has ceased.

The mutant strains had a small but variable impact on the morphology of host plants (Supplementary Figure 2). In contract to the results presented here, excessive hyphal branching by *E. festucae* mutants in plants almost always induces abnormalities in host morphology, such as stunted tillers (Takemoto et al., 2006, 2011; Tanaka et al., 2008; Eaton et al., 2010; Johnson et al., 2013b; Green et al., 2016). We assume that the *acyA* mutants did not stunt the host because hyphal distribution in the shoot apex was similar to the wild-type. The architecture of grasses is largely regulated by this tissue (where the leaves, inflorescences and tillers are differentiated) and competition for resources, hyphal overgrowth or disruption of host cell organization at this critical stage in development is likely to have a negative impact on host morphology. Since the distribution of hyphae in the shoot apex has not been investigated for many *E. festucae* mutants the discrepancy between hyphal branching and host morphology disturbance is unresolved.

Most fungi contain a single AC gene, however ancestral interspecific hybridizations between different *Epichloë* species have generated a number of strains with additional genomes (Schardl et al., 1994; Kuldau et al., 1999). Analyses of allopolyploids AR3046, and AR1006 (Craven et al., 2001; Moon et al., 2004) indicates that these strains each have two homeologs of *acyA*. A report describing the consequences of genomic and transcriptomics shock in a third relatively recent natural *Epichloë* hybrid (Lp1) suggests that both homeologs of most genes in the parental strains are also retained in this hybrid, and that there is little evidence for higher expression levels in one homeolog vs. the other (Cox et al., 2014). Similarly, in AR1006 one of the *acyA* genes was truncated and is likely a pseudogene, while in AR3046 both genes appear functional. Allopolyploids are often more competitive than their parental progenitors (Cox et al., 2014) and duplicate copies of key signaling pathway genes may, if both are retained, provide allopolyploid *E. festucae* strains (all asexual) resilience against mutations or allow for the evolution of new functions.

In summary, the cAMP cascade regulates saprotrophic growth of *E. festucae* in culture, with a role in enforcing the dominance of the hyphal apex and restricting development of lateral branches. Although ROS accumulation in *E. festucae* on PDA is positively regulated through cAMP, depletion of ROS alone cannot account for the hyper-branched phenotype in *E. festucae* Δ*acyA* as ROS synthesis mutants of *E. festucae* (Δ*racA* excepted) have a wild type phenotype when growing on PDA. In plants, wild-type *E. festucae* hyphal growth is tightly regulated and hyphal overgrowth is often observed in antagonistic *Epichloë*-*L. perenne* symbioses, such as those with disruptions in ROS synthesis (Takemoto et al., 2006; Tanaka et al., 2006). *E. festucae* Δ*acyA* mutants produce progressively more hyphae in host leaves as they age, presumably through the continued production of new hyphal tips that are normally restricted in wild type strains. Cyclic AMP is therefore critical in restricting potential overgrowth of the symbiont during colonization of developing host tissues, and in regulating the synchronicity in growth of the two organisms.

## Author contributions

CV, RJ, GB, LJ, and JK conceived the ideas for the study. All the authors participated in data analysis and interpretation, and contributed to the writing and editing of the manuscript. CV, MC, LJ, NF, SC, MG and WS contributed to the experimentation.

## Funding

This research was supported by Core Funding from AgResearch Ltd., and the New Zealand Ministry of Business and Primary Industries (to CV).

### Conflict of interest statement

The authors declare that the research was conducted in the absence of any commercial or financial relationships that could be construed as a potential conflict of interest.
