# Supplementary material for: cAMP Signaling Regulates Synchronised Growth of Symbiotic Epichloë Fungi with the Host Grass Lolium perenne
Source: Front Plant Sci. 2016 Oct 27;7:1546. doi: 10.3389/fpls.2016.01546 (PMC5082231; doi:10.3389/fpls.2016.01546)
Supplement: Supplementary file 1 [file DataSheet1.DOCX]

Supplementary Material

cAMP signalling regulates synchronised growth of symbiotic *Epichloë* fungi with the host grass *Lolium perenne*

**Christine R. Voisey^*^, Michael T. Christensen, Linda J. Johnson, Natasha T. Forester, Milan Gagic, Gregory T. Bryan, Wayne R. Simpson, Damien J. Fleetwood, Stuart D. Card, John P. Koolaard, Paul H. Maclean and Richard D. Johnson**

*** Correspondence:** Corresponding Author: christine.voisey@agresearch.co.nz

**Supplementary Table 1. Primers used in this study**

| **Primer** | **Sequence (5´-3´)** |
| --- | --- |
| AC5´-attB4 | GGGGACAACTTTGTATAGAAAAGTTGTCTACTCCGCCTTGGTAC |
| AC5´-attB1 | TGAAATCAGCGATATGCCCAAGTTTGTACAAAAAAGCAGTCCCC |
| AC3´-attB2 | GGGGACAGCTTTCTTGTACAAAGTGGTCAGAGATCGATCAAGAA |
| AC3´-attB3 | CAGCTGTATTCTTGGGCCCAACTTTATTATACAAAGTTGTCCCC |
| AcyA F | GCTCAGGATAGACAAAGGAGACGATAG |
| AcyA R | GTCGTCTGCCAACCCACCAT |
| AC SeqIntRev 2 | CTGAAAGCATCTGGGCACTC |
| AC M13For rev | CATTGTGCTGAACGGTTTTA |
| ACSEQrev4120 | CGAGGACGGCTGGCATCAG |
| ACSEQFOR3657 | CAAGATCGCCAAATACCTGCATG |
| AC5 F | tctactccgccttggtacttgataacc |
| AC3 R | GCCCAAGAATACAGCTGCTCTTATTG |
| ACKO F | ggattctggcctttcttcgaggc |
| ACKO R | TAGTTGTTGCCGCAATCCAGCAC |
| 1-1F | GTC CGA TCA TTC CAA GCT CG |
| 1-1R | TGG TGG GAA GTT CCC TGC AC |
| 138 F | CTGGCTGTGCAAATGATAATG |
| 2721 R | ATTACTCCGTACCTTGAGCTG |
| 2417F | CAGTAGAGTCTTGGAATGACG |
| 3964 R | GCACAAACTCGGTCACTTAC |
| 14671 F | CAGGATCGAGGCAAGTAAC |
| 15971 R | GTTTACTTGATCGACAGGACG |
| 15553 F | CTTTCCAGAGTTAAGCAGATGTC |
| 17683 R | CTGTACTTCTTCACCTCGC |

**Supplementary Table 2**

1. Percent identity between *acyA* genes recovered from allopolyploid strains and extant relatives of their predicted progenitor species. All genes appear to be capable of encoding functional proteins unless indicated otherwise (*). The *acyA* gene comparisons with highest identities are shaded.

| Strain | *acyA* accession | *E. bromicola*  AL0434 | *E. typhina* E8  ATCC 200736 |
| --- | --- | --- | --- |
| AR1006 | KT732647 | 99.9% | 95.1% |
|  | KT732648* | 94.8% | 97.3% |

| Strain | *acyA* accession | *E. bromicola*  AL0434 | *E. baconii*  ATCC 200745 |
| --- | --- | --- | --- |
| AR3046 | KT732649 | 97.2% | 99.9% |
|  | KT732650 | 98.9% | 97.2% |

1. Percent identity between *acyA* genes recovered from extant relatives of the predicted progenitor species of allopolyploids AR3046 and AR1006. All genes appear to be capable of encoding functional proteins. The percent identity between *acyA* genes from the same allopolyploid strains are shaded.

| Strain | *acyA* accession | AR1006  KT732647  *E. bromicola* | AR1006  KT732648*  *E. typhina* | AR3060  KT732649  *E. bromicola* | AR3060  KT732650  *E. baconii* |
| --- | --- | --- | --- | --- | --- |
| AR1006 | KT732647  *E. bromicola* |  |  |  |  |
|  | KT732648*  *E. typhina* | 94.8% |  |  |  |
| AR3046 | KT732649 *E. baconii* | 97.8% | 95.4% |  |  |
|  | KT732650  *E. bromicola* | 98.9% | 95.0% | 97.2% |  |

1. Percent identity between *acyA* genes recovered from extant relatives of the presumed progenitor species of allopolyploids AR3046 and AR1006. All genes appear to be capable of encoding functional proteins.

| Species | *E. bromicola*  AL0434 | *E. typhina* E8  ATCC 200736 | *E. baconii*  ATCC 200745 |
| --- | --- | --- | --- |
| *E. bromicola*  AL0434 |  |  |  |
| *E. typhina* E8  ATCC 200736 | 95.1% |  |  |
| *E. baconii*  ATCC 200745 | 97.2% | 95.6% |  |

**Supplementary Figure 1**

**
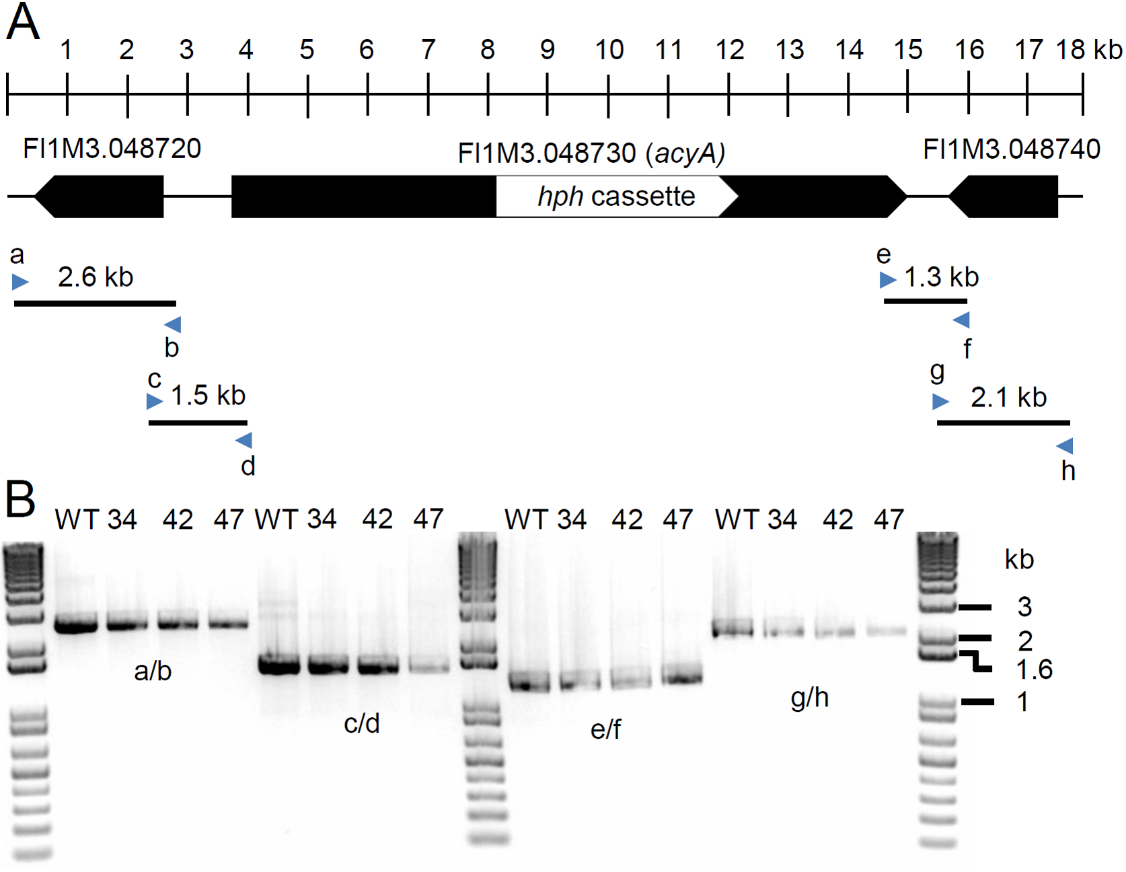
**

**Supplementary Figure 1.** **PCR of genes flanking the disrupted *acyA* gene in *E. festucae* Fl1 mutants.** A. Scale illustration of gene order and the coordinates of the primers used to confirm the integrity of the genes bordering the mutated *acyA* gene. The relevant gene model numbers from the *E. festucae* Fl1 genome (Version 2, 01-03-2011, Supercontig 14, 267100-280900 bp [17900 bp], [http://www.endophyte.uky.edu](http://www.endophyte.uky.edu/)) are listed above each gene. The *acyA* gene has an *hph* cassette insertion regulated through the GPDH promoter. Predicted PCR products from primer pairs a/b (138 F/2721 R), c/d (2417 F/ 3964 R), e/f (14,671 F/15,971 R) and g/h (15,553F/17,683 R) are shown. B. Agarose gel electrophoresis of PCR amplification products from genomic DNA of the WT and mutant strains ∆*acyA*34 (34), ∆*acyA*42 (42) and ∆*acyA*47 (47). The keys to the primer pairs used are shown below the DNA. The 1 kb^+^ DNA ladder (Invitrogen) was used as the size reference.

**Supplementary Figure 2**

**
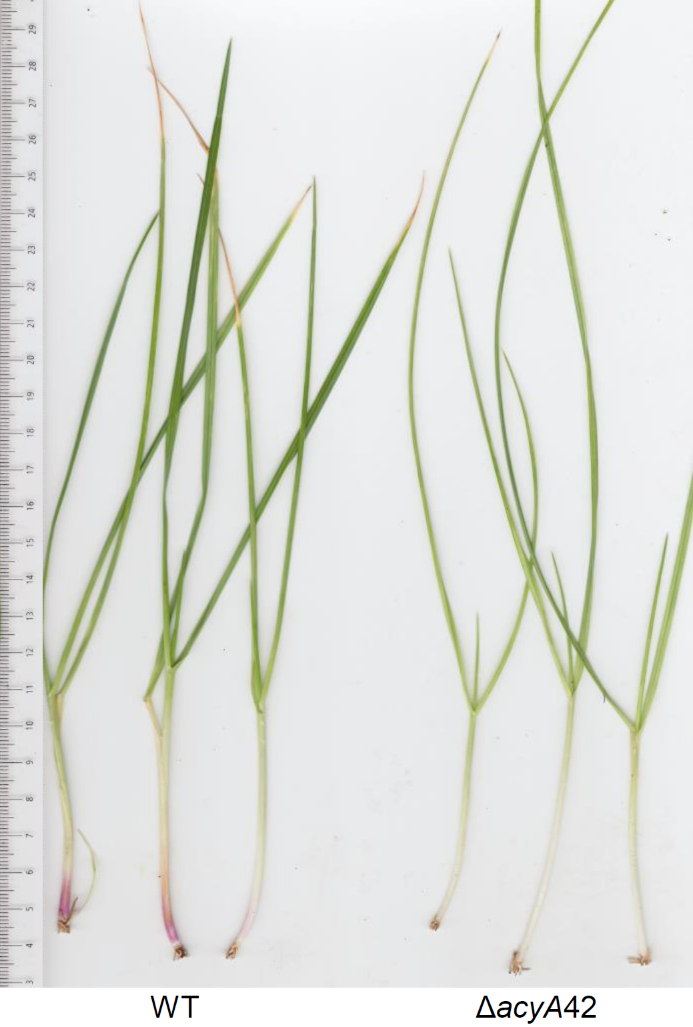
**

**Supplementary Figure 2. Effect of *acyA* disruption mutants on host phenotype.** Representative *L. perenne* tillers removed from plants infected with *E. festucae* Fl1 wild-type or Δ*acyA*42. The colonising endophytes were expressing EGFP. The same material was used to perform the CLSM studies presented in Figure 6.
